# Comparison of Structural Diagnosis and Management Approach Versus Myofascial Release for Plantar Heel Pain in People With Diabetes Mellitus: A Multicenter Randomized Clinical Trial Protocol

**DOI:** 10.1002/hsr2.72255

**Published:** 2026-04-02

**Authors:** Paroshmoni Biswas Mim, Suvro Nill Sarkar, Kazi Md Azman Hossain, Md. Feroz Kabir, Md. Zahid Hossain, Sharmila Jahan, Ehsanur Rahman, Abid Hasan Khan, K. M. Amran Hossain

**Affiliations:** ^1^ Department of Physiotherapy and Rehabilitation Jashore University of Science and Technology (JUST) Jashore Bangladesh

**Keywords:** diabetes mellitus, myofascial release, plantar heel pain, structural diagnosis management

## Abstract

**Background and Aims:**

Plantar heel pain (PHP) is prevalent among individuals with diabetes mellitus and contributes to chronic pain, reduced mobility, muscle weakness, and activity limitation. Conventional treatments such as medications and physical modalities are used but often lack long‐term effectiveness and cost‐efficiency. Physiotherapy approaches, including Structural Diagnosis and Management (SDM) and Myofascial Release (MFR), have demonstrated benefits; however, comparative evidence in diabetic populations is limited. This study aimed to evaluate and compare the effectiveness of SDM and MFR in reducing pain and disability and improving function in people with diabetes mellitus experiencing PHP.

**Methods:**

This study was a multicenter, assessor‐ and participant‐blinded randomized clinical trial. A total of 90 participants with diabetes mellitus, aged 30–65 years, and diagnosed with PHP were recruited from 3 diabetic hospitals in Bangladesh. Participants were randomly allocated (1:1) to the SDM group or the MFR group. Intervention was delivered over 8 weeks, comprising 24 sessions of 45–60 min each. The primary outcome, pain intensity, assessing by Visual Analog Scale (VAS). Secondary outcomes include ankle range of motion, muscle strength, and the Foot Function Index (FFI). The study adhered to CONSORT guidelines, and data analyze in SPSS using the intention‐to‐treat principle.

**Results:**

All primary and secondary outcomes were planned to assess at baseline, postintervention (8 weeks), and follow‐up (20 weeks) to examine both immediate and sustained effects of the interventions, with comparative analyses evaluating the effectiveness of SDM versus MFR in individuals with diabetes mellitus and PHP.

**Conclusions:**

This trial will provide the first direct comparison of SDM and MFR for the management of PHP in individuals with diabetes mellitus. The findings are expected to support evidence‐based, cost‐effective, nonpharmacological physiotherapy interventions and may inform future research and rehabilitation policies in diabetic foot care.

**Trial Registration:**

CTRI/2024/11/076311.

## Summary

- What is already known on this topic?
  ◦ Plantar heel pain is common among people with diabetes mellitus, causing pain, limited mobility, muscle weakness, and reduced function. Although various treatments are available, many lack long‐term effectiveness and value for money. SDM and MFR are specific, evidence‐based physiotherapy methods; however, there is limited comparative evidence on their efficacy in this population.
- What this study adds?
  ◦ This study presented a direct comparison between the SDM and MFR approaches in managing plantar heel pain in individuals with diabetes mellitus. This study assesses key clinical outcomes, pain intensity, ankle range of motion, muscle strength, disability, and activity limitation, over an 8‐week intervention period, followed by a 12‐week follow‐up. The study aims to establish evidence for a more effective and sustainable physiotherapy intervention, thereby improving treatment protocols in this population.
- How this study might affect research, practice, or policies?
  ◦ This study will identify the most effective intervention between SDM and MFR, promoting evidence‐based physiotherapy practices for managing PHP in diabetic patients. The findings may inform clinical decision‐making, contribute to the development of cost‐effective, nonpharmacological treatment guidelines, and influence future research directions and rehabilitation policies in diabetic foot care.

## 1 Introduction

Plantar heel pain (PHP) is the most common foot condition affecting the plantar region of the foot and is associated with surrounding structures, including the calf muscle, associated nerves, and plantar fascia, in individuals with a sedentary lifestyle or those who are active and maintain a prolonged standing or walking posture. Clinically, PHP can be associated with plantar fasciitis, typically characterized by discomfort, intermittent pain, and tenderness in the area of fascia located underneath the heel, extending toward the medial longitudinal arch (MLA) of the foot. There are two common clinical conditions associated with PHP, where plantar fasciitis is the most prevalent cause of PHP [1]. The thick band of connective tissue called plantar fascia, which consists of central, medial, and lateral bands. Where the central band arises from the medial tubercle of calcaneal bone toward toes, which provides support for the foot arch, that tissue becomes irritated and inflamed due to tensile overload or force.

Calcaneal spur is another cause. It is a fibromatosis that progressively infiltrates the plantar aponeurosis due to mechanical stress on the foot, gradually and continually increasing in the insertion point [2]. A heel spur is radiologically evident, and pain eventually extends from the heel to the lower extremities, worsening with walking or during the first minutes of rest. Painful ambulation leads to functional impairments. Moreover, this recurrent chronic condition hinders quality of life for an individual and presents with noteworthy disability [3].

The study of PHP incidence and prevalence is limited. It is estimated that 10% of individuals with lower limb pain can experience this condition in their lifespan [4, 5]. An epidemiological study in the United States has demonstrated that nearly one million individuals visit physicians annually for PHP [6]. Risk factors of PHP include obesity, decrease the arches, reduce flexibility, pes planus, improper footwear or hard surfaces of footwear, leading sedentary lifestyle, and prolong working posture in walking or standing barefoot on the hard surfaces or floors [7]. Strong association has been noted with PHP and metabolic diseases such as diabetes mellitus (DM), hyperuricemia, and hyperglycemia. The prevalence of PHP is higher among people with DM and diabetic foot (DF) [8, 9].

Systematic review between (1994 and 2013) reports, 4.5%–35.0% of Bangladeshi people have DM. The number might rise to 13.7 million by 2045 [10]. Approximately one million individuals with DM visit physicians for PHP [11]. Globally, 0.85% of diabetic people have PHP; among them, 1.31% have type‐2 and 0.92% have type‐1 DM [12]. The clinical presentation and biomechanical factors of PHP are similar in both nondiabetic and diabetic patients. However, in DM cases with PHP, elderly women with a high BMI are the most vulnerable group. In DM cases, degeneration of the plantar fascia and repetitive micro‐tears cause relapsing inflammation and delayed healing, creating a vicious cycle of injury and pain.

Shortening of the calf muscle leads to a series of biomechanical abnormalities, such as a decrease in the range of motion (ROM) of dorsiflexion and an increase in stress to the perifascial structures. Diagnosis of PHP can be made based on plantar fascia, heel spur, or myofascial abnormalities of the foot, according to the International Statistical Classification of Diseases and Related Health Problems (ICD) category. Clinical features are categorized according to specific diagnosis. Some clinical examination, such as the windlass test, longitudinal arch angle, tarsal tunnel syndrome test, and active and passive dorsiflexion ROM in the ankle joint, supports the diagnosis [13, 14].

Intervention guidelines for PHP in nondiabetic and diabetic patients are reported similarly in the previous clinical practice guidelines. Medications include anti‐inflammatory agents, such as NSAIDs. Many studies reported the significance of steroid injections in PHP in nondiabetic patients and some in diabetic patients to reduce inflammation, improve pain, and functional conditions. However, steroids have a long‐term consequence of tendon and osseous degeneration [13].

In physiotherapy practice, various interventions have been reported, including electrical modalities such as iontophoresis with dexamethasone 0.4% or acetic acid 5%, which give pain relief and improve function within 2–4 weeks. Manual therapy techniques such as nerve mobilization (effective within 1–3 months), soft tissue mobilization, passive neural mobilization, gliding of the ankle joint posteriorly, the subtalar joint laterally, and the first tarsometatarsal joint anterior‐posteriorly, and distraction manipulation of the subtalar joint are also commonly used to reduce pain and restore mobility for people with PHP [13]. Other conservative approaches include taping, orthotic devices, and night splints. Calcaneal taping may provide pain relief temporarily within 7–10 days. Orthotic devices, such as specially designed foot orthoses, reduce pain and improve function over a short‐term period of approximately 3 months. Night splints, available in sock‐type, anterior, or posterior designs, are recommended for individuals with PHP lasting 6 months or more. They are typically worn for 1–3 months [15].

Studies on PHP in nondiabetic patients have revealed that Myofascial Release (MFR) to the calf muscles and plantar fascia is a practical manual therapy technique [16]. Recent studies indicate that the Structural Diagnosis and Management (SDM) approach—targeting calf muscles, plantar fascia, lower limb muscles, and myoneural structures—offers short‐ to intermediate‐term pain relief and improved functional independence in people with PHP. However, PHP is higher in DM cases and has a poor prognosis, and there is a significant research gap addressing effective manual therapy approaches for PHP in people with DM [17, 18, 19, 20, 21].

Therefore, the primary goal of this study is to compare the effectiveness of two manual therapy approaches, SDM and MFR, in treating individuals with PHP who have DM. The objectives are (1) to determine baseline compatibility of both groups in sociodemographic and clinical variables; (2) to measure between‐group and within‐group posttreatment outcomes in pain, ankle ROM, muscle strength, activity limitation, and disability; and (3) to compare the follow‐up outcomes of both groups. After 8 weeks of interventions, the SDM or MFR group is expected to show better improvement in PHP for people with DM.

## 2 Methods

### 2.1 Study Design

**Figure 1:**
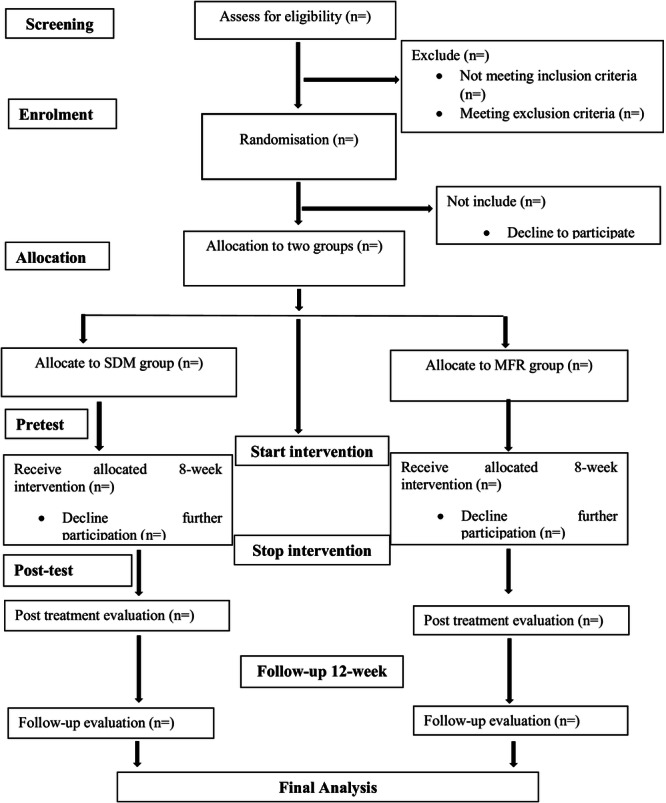
CONSORT flow diagram.

This study was a multicenter, parallel, assessor‐ and participant‐blinded randomized clinical trial with an 8‐week intervention period, conducted at the Nurul Islam Diabetic Centre, Ibn Sina Diabetes Centre, and City Hospital & Diabetic Care, Jashore, Bangladesh, from September 2025 to March 2026. Eligible participants were randomly assigned in a 1:1 ratio to either the SDM or MFR group. This trial has been designed following the Standard Protocol Items: Recommendations for Interventional Trials (SPIRIT) guidelines, which offer a comprehensive framework for developing RCT protocols, as detailed in Table 1. Additionally, the Consolidated Standards of Reporting Trials (CONSORT) statement has been adopted to improve the quality and completeness of RCT reporting, as shown in Figure 1.

**Table 1:** SPIRIT table.

Timepoint	Enrollment	Allocation		Post‐allocation	
	−*T*_1_	*T*_0_	*T*_1_	*T*_2_	*T*_3_
*Enrollment*					
Eligibility screening	X				
Informed consent		X			
Demographic and clinical assessment			X		
Group allocation		X			
*Intervention*					
SDM Group			X	X	
MFR Group			X	X	
*Assessment*					
Pain intensity			X	X	X
Range of motion			X	X	X
Muscle strength			X	X	X
Foot Function Index			X	X	X

*Note:* −*T*_1_, prestudy enrolment; *T*_0_: group allocation; *T*_1_, baseline before intervention; *T*_2_, measurement after 8 weeks; *T*_3_, measurement after 20 weeks.

Abbreviations: MFR, myofascial release; SDM, Structural Diagnosis and Management.

### 2.2 Sample Size Calculation

The required sample size was calculated using G*Power software version 3.1.9.7 (University of Kiel, Kiel, Germany). Based on an effect size of 0.50, with a significance level (*α*) of 0.05 and statistical power of 0.80, a total of 72 participants were required for the study. To assume a potential 25% dropout rate, the final adjusted sample size was increased to 90 participants, with 45 allocated to each group [3].

### 2.3 Participant Recruitment and Screening

Between September 2025 and March 2026, DM with PHP were recruiting from three hospitals in Jashore, Bangladesh. Participants were screened for eligibility by two trained research assistants, and those who met the criteria provided written informed consent. Following CONSORT guidelines, participants were randomly assigned to their allocated groups using a concealed allocation method. Four blinded assessors conducted baseline assessments prior to the intervention's commencement. The intervention follows a standardized, concealed protocol, with participants blinded to group allocation to minimize bias.

### 2.4 Eligibility Criteria

Participants were screened for eligibility by trained research assistants. Inclusion criteria: (1) age between 30 and 65 years for both male and female [17]; (2) participants diagnosed with unilateral or bilateral heel pain or plantar fasciitis (according to ICD‐10 criteria, ICD diagnosis codes M72.2); (3) localized pain in the plantar region of the foot when placed their barefoot on the floor in the morning; (4) ankle dorsiflexion is more limited than plantar‐flexion; and (5) suffering with PHP from more than 4 weeks [17]. Exclusion criteria: (1) confirmation and diagnosis of diabetic peripheral neuropathy; (2) history of lower extremity fracture, rheumatoid arthritis, osteoarthritis, carcinoma, surgery of the foot, and metal implant around ankle [15]; (3) active foot ulceration, infection, severe peripheral vascular disease, gangrene of the foot, cyanotic symptom, and severe neurological condition [11]; (4) contraindicated to manual therapy or exercise intervention [17]; and (5) participants taken corticosteroids injection within 6 months [16].

### 2.5 Randomization and Blinding

Eligible participants with written informed consent were randomly allocated to their respective groups, either the SDM or the MFR group. The randomization sequencing process was conducted using the “Rand” function in Microsoft Excel 2013 software. A sequential list of participants' IDs, sorted according to the randomly generated numbers. This procedure helps to prevent bias. Participants were allocated to a parallel intervention group in a 1:1 ratio. This is an assessor‐ and participant‐blinded randomized clinical trial, with outcome assessors and participants blinded to group allocation. Physiotherapists delivering manual therapy could not be blinded, but both groups received identical conventional physiotherapy to control for nonspecific effects. Therapists were trained to follow standardized protocols and avoid discussing treatment or outcomes with participants or assessors to minimize bias. Inter‐rater reliability for manual techniques will be established, and any unblinding will be documented and reported.

### 2.6 Interventions

Both groups undergo an 8‐week intervention program, consisting of three sessions per week, with each session lasting 45–60 min, following the designed treatment protocol.

The SDM group receives manual therapy based on the SDM approach for PHP. This technique targets the calf muscles using graded interventions classified by pressure velocity and biomechanical positioning, combined with stretching of myoneural structures [17]. The MFR group receives MFR therapy, involving deep stripping of the plantar fascia and targeted surface points of the calf muscles, following a standard protocol [18]. Both groups receive designed conventional physiotherapy treatment including ankle strengthening exercise, therapeutic ultrasound, and ice compressions [18, 19, 20, 21, 22]. Detailed descriptions of the interventions for both groups are provided in Table 2.

**Table 2:** Intervention details.

Exercise	Description	Dosages
*Structural Diagnosis and Management Approach (SDM)*		
Calf muscle release [17]	Prone position: Superior to inferior; Lateral to medial Or, Supine position: Release with rolling techniques	10 repetitions for each direction/technique; 1–3 sets Application of gentle‐firm‐deep pressure
Calf muscle stretching in ankle dorsiflexion position [17]	Stretching of both the gastrocnemius and the soleus in different positions Application of one‐third to two‐thirds of the full dorsiflexion technique	10 repetitions; 15 s' hold; 1–3 sets
Myoneural stretching [17]	Hip and knee flexed to 90° Modified straight leg raise with ankle dorsiflexion	3–5 repetitions; 15 s' hold; 3 sets Repetition increases progressively
*Myofascial Release Approach (MFR)*		
Deep stripping [18]	Plantar fascia → Calcaneus Calf muscles Deep posterior leg compartment	Each 5–7 repetitions; 15–30 s' hold; 1–3 sets Application of gentle‐firm‐deep pressure
*Conventional Physiotherapy for Both Groups*		
Ankle strengthening exercise [19, 22]	Resistance exercise (dorsiflexion and plantarflexion with resistance band); towel crunch; heel raise	10 reps; 10 s hold; 1–3 sets
Therapeutic ultrasound [20]	Application of therapeutic pulse ultrasound	Intensity of 1.8 W/cm^2^ and frequency of 1 MHz; 5 min
Ice compression [21]	To reduce pain and prevent inflammation Use an ice cube and an ice pack	8 min

*Note:* Applicable for every exercise: 15 s of rest between each set, and 1 min of rest between different modes of exercise.

The duration and frequency of sessions may be adjusted individually based on each participant's condition. To ensure accurate tracking, a monitoring sheet is maintaining for each participant attending every session.

### 2.7 Outcome Measurements

Baseline sociodemographic and clinical characteristics were collected immediately after obtaining informed consent from participants and before the initiation of the intervention. All primary and secondary outcomes were planned to analyze at three time points: baseline (Week 0), posttest (Week 8), and follow‐up (Week 20), by four trained and blinded assessors.

### 2.8 Sociodemographic and Clinical Characteristics

The researcher developed a structured questionnaire to collect sociodemographic and clinical characteristics from participants. This includes personal details such as age, gender, and occupation; demographic information; and condition‐related variables such as BMI, type and duration of DM, radiological confirmation of diagnosis, duration of PHP, affected foot, type of footwear, and duration of footwear use.

### 2.9 Primary Outcome

#### 2.9.1 Pain Intensity

Pain intensity was measured using a 10 cm Visual Analog Scale (VAS), a widely accepted tool for evaluating pain. The scale consists of a 10 cm horizontal line anchored at 0 (“no pain”) and 10 (“severe pain”). The VAS demonstrates excellent test–retest reliability (ICC = 0.99; 95% CI: 0.989–0.992) and strong validity [23].

### 2.10 Secondary Outcomes

#### 2.10.1 Range of Motion (ROM)

Participants actively perform dorsiflexion and plantarflexion using a digital dynamometer (ActiveForce‐2), with measurements recorded in degrees via the device's app. A study reported excellent reliability for the portable dynamometer, with ICC values of 0.972 for dorsiflexion and plantarflexion. Additionally, strong reliability was found between right and left ankle measurements: plantarflexion ICC = 0.89 and dorsiflexion ICC = 0.94 [24, 25].

#### 2.10.2 Muscle Strength

A portable digital hand dynamometer (ActiveForce‐2) and its companion app is used to assess isometric strength of the dorsiflexors and plantarflexors of the affected foot, with measurements recorded in kilogram‐force (kgf). Previous research has demonstrated that portable dynamometers exhibit high inter‐rater and test–retest reliability, with strong correlations for dorsiflexion and plantarflexion strength (*r* = 0.827, *r* = 0.973) [25].

#### 2.10.3 Disability and Activity Limitation

The Foot Function Index (FFI) is used to evaluate overall pain, disability, and activity limitation. This self‐administered questionnaire comprises 23 items across 3 subscales: pain (9 items), disability/function (9 items), and activity limitation (5 items). Previous studies have reported test–retest reliability ranging from 0.69 to 0.87 and internal consistency between 0.73 and 0.96, alongside strong correlations with clinical measures of foot pathology, confirming its validity as a standard assessment tool. FFI scores were planned to analyze at both the total and subscale levels, with measurements taken at baseline, postintervention, and follow‐up. Between‐group and within‐group comparisons will be performed for each subscale to provide a domain‐specific interpretation of treatment effects [26, 27].

### 2.11 Study Procedure

After screening by trained research assistants, eligible participants were thoroughly informed about the study's objectives and procedures, and then obtain their written informed consent. Once screened, participants were randomly assigned to their allocated groups using a predetermined randomization sequence to ensure unbiased allocation. Four blinded assessors collected baseline data at the study centre using an ethically approved questionnaire designed specifically for this research, which includes sociodemographic information and clinically relevant measures to confirm baseline compatibility with no statistically significant differences between groups. Following the baseline assessment, participants receive their assigned intervention for 8 weeks. After this period, blinded assessors will perform posttest (Week 8) and follow‐up (Week 20) evaluations by measuring primary and secondary outcome variables. This entire study procedure strictly following the SPIRIT 2013 guidelines (Table 1), ensuring methodological rigor and ethical compliance throughout the study.

### 2.12 Safety Measures and Adverse Reaction Management

Although no adverse events are anticipated from the treatment, the monitoring team remains vigilant and promptly notifies the appropriate healthcare professionals of any unexpected occurrences that may arise during or after the intervention. All treatment procedures are thoroughly documenting in daily SOAP notes to ensure clear records and to prevent any future misunderstandings or complaints from participants. Before initiating the intervention, the physiotherapist carefully screens for any contraindications. If participants experience any discomfort, pain, or skin irritation after the treatment, they should immediately report these symptoms to both the physician and physiotherapist. Identified any harmful effects, which will be transparently reported by the researcher in the final publication. Additionally, an adverse event reporting checklist is used throughout the intervention period to facilitate systematic monitoring.

### 2.13 Data Management

After each assessment, assessors verify the data daily to ensure accuracy. The final study data set will be accessible only to the study manager, principal investigators, and data auditors. Upon completion of the study, all authors will have equal access to anonymized data. The principal investigator will securely store all hard and electronic copies of the collected data, ensuring that no access is granted to identifiable participant information. Manual data entries will undergo double‐checking to minimize errors. Online data will be carefully stored on a password‐protected server managed by the postgraduate program in Physiotherapy and Rehabilitation. Each participant is assigned a unique identification number, and data will be encoded securely using this ID. The ID number will be encrypted separately from any identifying information to maintain participant confidentiality throughout the study.

### 2.14 Data Monitoring

Two independent individuals were appointed to monitor and audit group enrollment, the intervention protocol, and any adverse effects experienced by participants. They are not directly included in this study. These monitors have access to review data and conduct interim analyses. Any changes to the research or treatment protocol are promptly communicated by the principal investigator to the Ethical Review Board.

### 2.15 Data Analysis

Data analysis will be performed by an independent statistician using SPSS version 22, with anonymized and encrypted data to ensure confidentiality. Prior to analysis, data will be screened for completeness and outliers. The parametricity of the data will be assessed using the Kolmogorov–Smirnov and Shapiro–Wilk tests, supported by evaluation of skewness and kurtosis and visual inspection of data distribution through histograms and normal probability plots. Descriptive statistics, including mean and standard deviation for normally distributed variables, median and interquartile range for non‐normally distributed variables, and frequency and percentage for categorical variables, will be used to summarize continuous and categorical data. Baseline characteristics, including body mass index and duration of diabetes, will be reported to assess group comparability, and exploratory subgroup analyses for high‐risk participants will be considered if clinically relevant patterns are observed. For comparisons across three time points (baseline, postintervention, and follow‐up), repeated‐measures ANOVA will be applied for normally distributed data, with the Friedman test used as the nonparametric alternative. Between‐group and within‐group differences will be analyzed using independent and paired *t*‐tests for parametric data, or Mann–Whitney *U* and Wilcoxon signed‐rank tests for nonparametric data, as appropriate. The laterality of PHP (unilateral or bilateral) will be recorded and incorporated into the analysis to minimize potential confounding effects on ROM and muscle strength outcomes. Effect estimates and mean differences will be reported with 95% confidence intervals alongside *p* values. All statistical tests will be two‐tailed, with statistical significance set at *p* < 0.05, *p* < 0.01, and *p* < 0.001. In addition, baseline, posttest, and follow‐up change scores will be evaluated, and an intention‐to‐treat analysis using the last observation carried forward method will be applied to handle missing data and preserve the validity of the analysis.

### 2.16 Dissemination

The results of this study will be submitted for publication to peer‐reviewed journals. Initially, a seminar will be organized to present the findings to physiotherapists, researchers, and healthcare professionals. Following this, a training and knowledge‐sharing session will be conducted specifically for physiotherapists to enhance their understanding of the treatment approach. Disseminating research findings is a crucial part of the research process, facilitating knowledge exchange among researchers, clinicians, healthcare providers, and broader audiences. This process also helps identify gaps for future research. Finally, the study's results will be presented at national and international conferences worldwide.

## 3 Discussion

The primary aim of this study is to evaluate the effectiveness of the SDM Approach versus MFR in treating PHP in diabetic patients. Previous research indicates that approximately one million individuals are referred annually to a doctor for a definitive diagnosis of plantar fasciitis. A recent cohort study identified the prevalence of plantar fasciitis as 720,000, with an incidence of 0.85% among diabetic patients [8, 9, 10, 11, 12]. Several studies have explored various interventions that could provide interim relief from PHP, plantar fasciitis, and heel spurs, such as considering and providing anti‐inflammatory agents, electrical modalities, and manual therapies like nerve mobilization, often focusing solely on the central pain area rather than the affected structures [14, 15].

However, our study considers the potential scope by including the associated structures, which may be a more critical factor in achieving sustainable outcomes. Without addressing these, diabetic patients are increasingly at risk of this foot condition, which often remains untreatable and misunderstood. To date, no research has directly compared and analyzed yet the effectiveness of SDM versus MFR in treating PHP in people with diabetes mellitus. The long‐term objective of this study is to play a vital role in the management of this global condition.

This study offers several significant benefits. It marks the first attempt in Bangladesh to compare the effectiveness of SDM and MFR in managing PHP among individuals with diabetes mellitus, incorporating a 12‐week follow‐up period. The study highlights the potential contribution of these intervention strategies not only in reducing pain but also in improving functional outcomes that enhance patients' quality of life. By addressing a significant gap in existing research, this study may offer valuable insights for clinical practice and future research in DF management.

However, limitations include the focus on a specific area in Bangladesh, which may restrict the broader applicability of its findings. Additionally, the presence of multiple comorbidities and a limited sample size are notable constraints. Future research should involve a larger and more diverse population, with an extended follow‐up duration. It is also recommended that subsequent studies adopt more rigorous research designs, suited to resource‐limited settings, to enhance the validity and generalizability of the findings.

## 4 Study Status

This randomized clinical trial is currently recruiting participants and is expected to be completed within the preplanned timeline.

## Author Contributions

Conceptualization: Paroshmoni Biswas Mim and K.M.A.H. (Corresponding Author). Methodology: Paroshmoni Biswas Mim, K.M.A.H., and K.M.A.H. (Corresponding Author). Software: Md. Feroz Kabir and Md. Zahid Hossain. Investigation: Suvro Nill Sarkar and Sharmila Jahan. Writing – original draft: Paroshmoni Biswas Mim, K.M.A.H., Abid Hasan Khan, and K.M.A.H. (Corresponding Author). Writing – review and editing: Paroshmoni Biswas Mim, Suvro Nill Sarkar, K.M.A.H., Md. Feroz Kabir, Md. Zahid Hossain, Sharmila Jahan, Ehsanur Rahman, Abid Hasan Khan, and K.M.A.H. (Corresponding Author). Visualization: Ehsanur Rahman and K.M.A.H. Supervision: K.M.A.H. (Corresponding Author). Project administration: K.M.A.H., Md. Feroz Kabir, Md. Zahid Hossain, Sharmila Jahan, Ehsanur Rahman, and K.M.A.H. (Corresponding Author). All authors have read and approved the final version of the manuscript. Corresponding and Guarantor: K. M. Amran Hossain has full access to all of the data in this study and takes complete responsibility for the integrity of the data and the accuracy of the data analysis.

## Ethics Statement

This study received ethical approval from the Institutional Review Board, Department of Physiotherapy and Rehabilitation, Jashore University of Science and Technology (Approval ID: PTRJUST/IRB/2025/03/192411). It is also prospectively registered with the Clinical Trial Registry India (CTRI) (Registration ID: CTRI/2024/11/076311). The study is following the 2020 Declaration of Helsinki. Participant rights will also be protected in accordance with the World Medical Association's ethical guidelines.

## Consent

Participant consent is being obtained for the publication and dissemination of study findings.

## Conflicts of Interest

The authors declare no conflicts of interest.

## Supporting information

Supporting File 1.

Ethical Approval Letter.

Patient Consent Form.

Funding Letter.

## Data Availability

No data are available as no data sets were generated or analyzed for this multicenter randomized clinical trial protocol. After completing the final results of this study, the data sets of the participants and the statistical analysis will be made available to the corresponding author upon reasonable request.
