# Supplementary material for: Comparison of Structural Diagnosis and Management Approach Versus Myofascial Release for Plantar Heel Pain in People With Diabetes Mellitus: A Multicenter Randomized Clinical Trial Protocol
Source: Health Sci Rep. 2026 Apr 2;9(4):e72255. doi: 10.1002/hsr2.72255 (PMC13052310; doi:10.1002/hsr2.72255)
Supplement: Supplementary file 4 — Funding Letter. [file HSR2-9-e72255-s004.pdf]

Dept. of Physiotherapy and Rehabilitation
Jashore University of Science and Technology
Jashore -7408, Bangladesh
Phone: +88 0242142160
Fax: +88 0421 61199, 62238
E-mail: chairmanphysio@gmail.com

ফিজিওথেরাপি অ্যান্ড রিহ্যাবিলিটেশন বিভাগ
যশোর বিজ্ঞান ও প্রযুক্তি বিশ্ববিদ্যালয়
যশোর-৭৪০৮, বাংলাদেশ।
ফোন: +৮৮ ০২৪২ ১৪২১৬০
ফ্যাক্স: +৮৮ ০৪২১ ৬১১৯৯, ৬২২৩৮

Reference No: PTR/2025/69 **Date: March 18, 2025**

FUNDING APPROVAL LETTER

Paroshmoni Biswas Mim
Student
Department of Physiotherapy and Rehabilitation
Jashore University of Science and Technology
Jashore-7408, Bangladesh.

Dear Paroshmoni Biswas Mim,

I am pleased to inform you that the academic committee recently voted for a grant to your study of BDT 200,000.00 (USD 1682 Approx.), which will be released this year. The purpose of this grant is to assist in the data collection and intervention provision for the project titled "Comparison of Structural Diagnosis and Management (SDM) Approach versus Myofascial Release (MFR) for Plantar Heel Pain in People with Diabetes Mellitus: A Multicenter Randomized Clinical Trial."

In all public acknowledgements, we prefer that you indicate this grant as being received from the University Grants Commission of Bangladesh through Jashore University of Science and Technology. The recipients must adhere to the existing taxation policy of the Government of the People's Republic of Bangladesh.

We ask that you acknowledge receipt of this grant and indicate your acceptance of the terms discussed above. The grant was approved on March 16, 2025. The grant number is 23-FoHS-10. Please include this number in the acknowledgement and funding information sections of your reports and publications. You also have to submit a detailed report in April 2026 to the Department of Physiotherapy and Rehabilitation regarding your research project progression, in terms of receiving the allocated budget. In the meantime, we wish you our very best for continued success.

Sincerely,

Dr. Md. Zahid Hossain
Assistant Professor and Chairman
Department of Physiotherapy and Rehabilitation
Jashore University of Science and Technology (JUST), Jashore-7408, Bangladesh
E-mail: mz.hossain@just.edu.bd

ফিজিওথেরাপি অ্যান্ড রিহ্যাবিলিটেশন বিভাগে ফিজিওথেরাপি চিকিৎসা ব্যবস্থা চলমান রয়েছে।
স্বল্প মূল্যে যে কেউ উক্ত বিভাগে ফিজিওথেরাপি চিকিৎসা নিতে পারবেন।
রোগী দেখার সময়: প্রতি শনি-বুধ (সরকারি ছুটি ব্যতিত), সকাল-৯টা থেকে বিকাল-৫টা পর্যন্ত।
যোগাযোগ: এম আর খান মেডিকেল সেন্টার (৩য় তলা), রুম নং-৩১১, মোবাইল: ০১৫৫৩ ৩৩৭ ০৯৪ ।
